# Endothelial to Mesenchymal Transition Represents a Key Link in the Interaction between Inflammation and Endothelial Dysfunction

**DOI:** 10.3389/fimmu.2018.00294

**Published:** 2018-02-20

**Authors:** Jin Gu Cho, Aram Lee, Woochul Chang, Myeong-Sok Lee, Jongmin Kim

**Affiliations:** ^1^Division of Biological Sciences, Sookmyung Women’s University, Seoul, South Korea; ^2^Department of Biology Education, College of Education, Pusan National University, Busan, South Korea

**Keywords:** endothelial dysfunction, inflammatory process, endothelial to mesenchymal transition, endothelial heterogeneity, vascular disease

## Abstract

Endothelial cells that line the inner walls of blood vessels are in direct contact with blood and display remarkable heterogeneity in their response to exogenous stimuli. These ECs have unique location-dependent properties determined by the corresponding vascular beds and play an important role in regulating the homeostasis of the vascular system. Evidence suggests that vascular endothelial cells exposed to various environments undergo dynamic phenotypic switching, a key biological program in the context of endothelial heterogeneity, but that might result in EC dysfunction and, in turn, cause a variety of human diseases. Emerging studies show the importance of endothelial to mesenchymal transition (EndMT) in endothelial dysfunction during inflammation. EndMT is a complex biological process in which ECs lose their endothelial characteristics, acquire mesenchymal phenotypes, and express mesenchymal cell markers, such as alpha smooth muscle actin and fibroblast-specific protein 1. EndMT is induced by inflammatory responses, leading to pathological states, including tissue fibrosis, pulmonary arterial hypertension, and atherosclerosis, *via* dysfunction of the vascular system. Although the mechanisms associated with inflammation-induced EndMT have been identified, unraveling the specific role of this phenotypic switching in vascular dysfunction remains a challenge. Here, we review the current understanding on the interactions between inflammatory processes, EndMT, and endothelial dysfunction, with a focus on the mechanisms that regulate essential signaling pathways. Identification of such mechanisms will guide future research and could provide novel therapeutic targets for the treatment of vascular diseases.

## Introduction

Endothelial cells (ECs) play a key role in maintaining vascular homeostasis in response to various stimuli. They can regulate vascular tone, permeability, coagulation, and inflammation through the regulation of numerous mediators, such as endothelium-derived relaxing and contracting factor, cell-adhesion molecules, cytokines, and chemokines (1, 2). However, vascular injuries resulting from procedures and conditions, such as angioplasty, stenting, diabetes, hypertension, and immune-mediated damage, can lead to endothelial dysfunction, resulting in disturbance or loss of normal endothelial functions (1, 3–5). Many studies have revealed an association between endothelial dysfunction and inflammatory stress in vascular biology. Under conditions of chronic inflammation, sustained activation of ECs by inflammatory stimuli, such as interleukin (IL)-6, tumor necrosis factor-α (TNF-α), IL-1β, and pathogens, cause alterations in normal endothelial function, resulting in impaired endothelial-dependent immune response, which is the hallmark of endothelial dysfunction (6–9). Indeed, endothelial dysfunction due to inflammatory stress contributes to the pathogenesis of many diseases, including fibrosis, atherosclerosis, pulmonary arterial hypertension (PAH), and pathological angiogenesis (10–17). In addition, emerging evidence shows that the nucleotide-binding domain, leucine-rich-containing family, pyrin domain-containing-3 (NLRP3) inflammasome not only has a role as a critical sensor in immune response, but also has a critical role in endothelial dysfunction and the pathogenesis of vascular diseases, such as atherosclerosis and metabolic syndrome (18–20). Assembly and activation of the NLRP3 inflammasome results in the conversion of the inactive procaspase-1 into active caspase-1, with subsequent secretion of mature IL-1β and IL-18 in such diseases (21–25).

Accumulating evidence suggests that endothelial to mesenchymal transition (EndMT) represents a key link in the complex interactions between inflammatory stress and endothelial dysfunction. EndMT is a phenotypic switching process by which ECs lose their characteristics and acquire mesenchymal traits (26, 27). EndMT exhibits features similar to those of epithelial to mesenchymal transition (EMT) and is often considered a specific form of EMT (26, 28). Although both processes use the same signaling pathways and result in cells with a mesenchymal phenotype, studies of the differences between EMT and EndMT are needed due to differences in the origin, fundamental function, and microenvironment of ECs and epithelial cells (29). EndMT was first discovered and has been studied in heart development and emerging studies show that EndMT can occur in postnatal pathologies associated with several diseases, such as fibrosis, cancer, neointima formation, cerebral cavernous malformations, atherosclerosis, and PAH (26, 28–30). Indeed, it has been reported that EndMT contributes to endothelial dysfunction during inflammatory conditions, and that some inflammatory mediators, such as IL-1β, TNF-α, nuclear factor kappa B (NF-κB) transcription factor, and endotoxins, can activate ECs and convert them to mesenchymal-like cells through the EndMT process (6, 7, 31). However, how EndMT contributes to disease progression remains unclear (32), and the specific role of EndMT in inflammatory stimulus-induced endothelial dysfunction has not been fully elucidated due to the dynamic nature of the EndMT process, which consists of multiple steps.

A single layer of ECs lining blood vessels displays heterogeneity in function, morphology, gene expression, and antigen composition depending on location (2) and behaves differently based on its exposure to different microenvironments (33). Therefore, it is also important to understand the molecular basis of inflammation-induced EndMT in the context of endothelial heterogeneity, because this can be critical for developing personalized vascular therapies for patients with vascular bed specific diseases (33).

In this review, we summarize the knowledge currently available regarding the role of EndMT in inflammatory processes and discuss endothelial heterogeneity in the context of inflammation.

## ENDMT Mediators and Signaling Pathways During Inflammation

Endothelial cells play an important role in the maintenance of homeostasis across the entire vascular system (10, 34). ECs actively participate in the regulation of immune responses to various stimuli. To this end, the inflammation-mediated signaling pathway has been extensively studied (6, 17). However, cell signaling associated with inflammation-induced EndMT remains poorly understood. Nevertheless, the molecular mechanisms underlying inflammation-induced EndMT have been gradually identified based on observations of EMT processes that are relatively well studied on inflammatory responses (6). Current evidence suggests that inflammation-induced EndMT, similar to that of EMT, is largely governed by two signaling pathways: the transforming growth factor beta (TGFβ) pathway and the non-TGFβ pathway (35). TGFβ is the most well-known EndMT inducer and upregulates the expression of transcription factors, such as snail, slug, and zinc finger E-box-binding homeobox 1 (ZEB1). These transcription factors then upregulate the expression of mesenchymal markers, such as alpha smooth muscle actin (α-SMA), smooth muscle protein 22 alpha (SM22α), calponin, vimentin, type I collagen, fibronectin, fibroblast-specific protein 1 (FSP-1), N-cadherin, matrix metalloprotein (MMP)-2, and MMP-9 (6, 36, 37).

**Figure 1:**
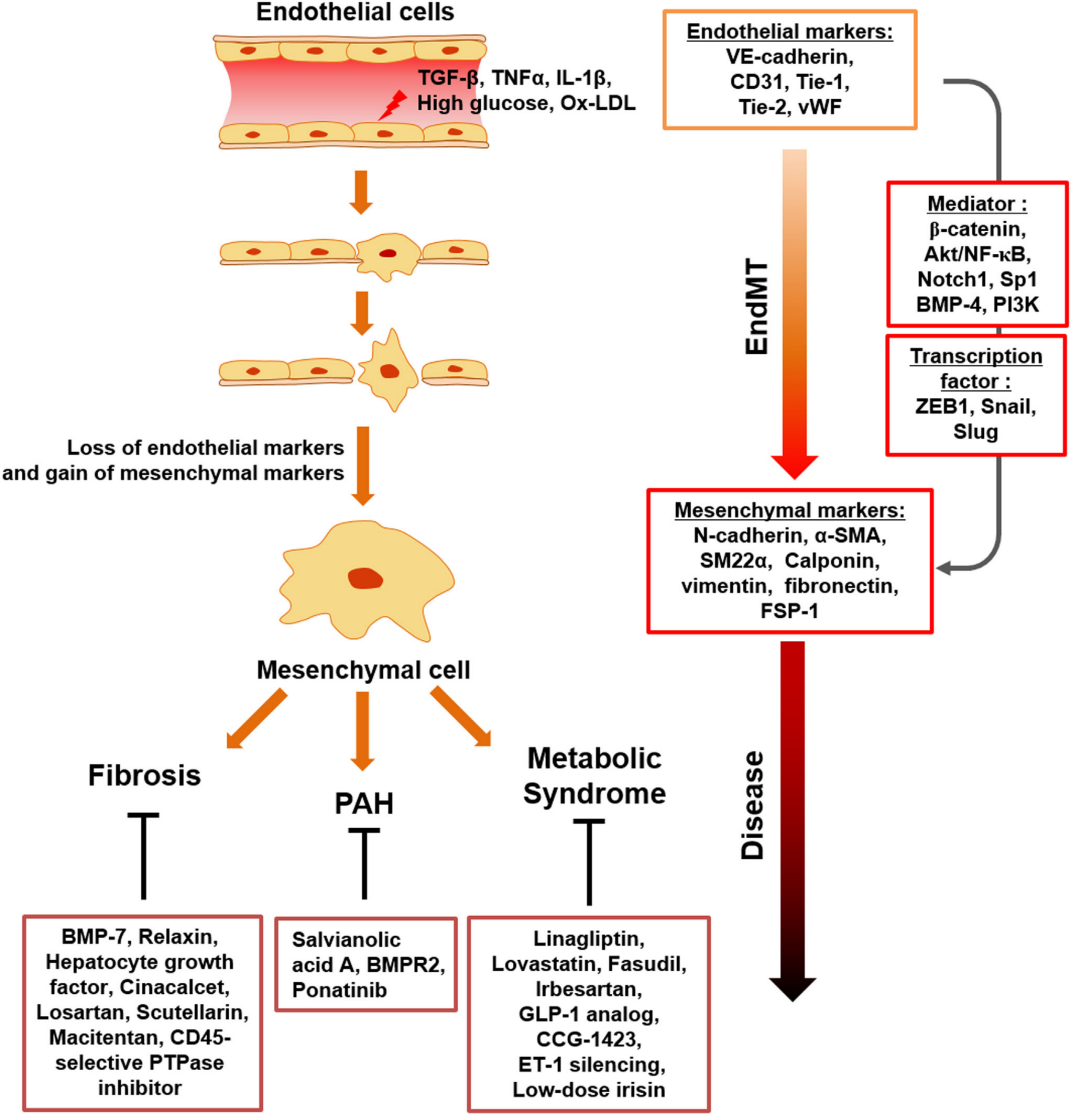
Schematic representation of endothelial to mesenchymal transition (EndMT) transition in response to inflammatory stimuli and metabolic dysfunction. Upon chronic inflammatory conditions, involving tumor necrosis factor-α (TNF-α), transforming growth factor beta (TGFβ), IL-1β, and endotoxin and metabolic dysfunction, such as increased serum LDL, glucose, diverse ECs undergo activation, which results in loss of endothelial cell markers and acquisition of mesenchymal-cell markers. EndMT contributes to endothelial dysfunction under inflammatory conditions and metabolic dysfunction, with EndMT mediators identified. This process can cause a variety of postnatal diseases, such as fibrosis, PAH, and metabolic syndrome.

It has been identified that EndMT related to direct immune responses is triggered in response to pro-inflammatory cytokines, such as TNF-α, IL-1β, and their combinations. Similarly, inflammation-induced EndMT is characterized by the loss of endothelial phenotypes and gain of mesenchymal-like characteristics, and endothelial/mesenchymal markers are tightly controlled by EndMT mediators, such as ZEB1, β-catenin, Akt/NF-κB, snail, slug, Notch1, bone morphogenetic protein (BMP)-4, Sp1, phosphoinositide 3-kinase (PI3K), and enhancer of zeste homolog 2 (EZH2) (Figure 1).

**Figure 2:**
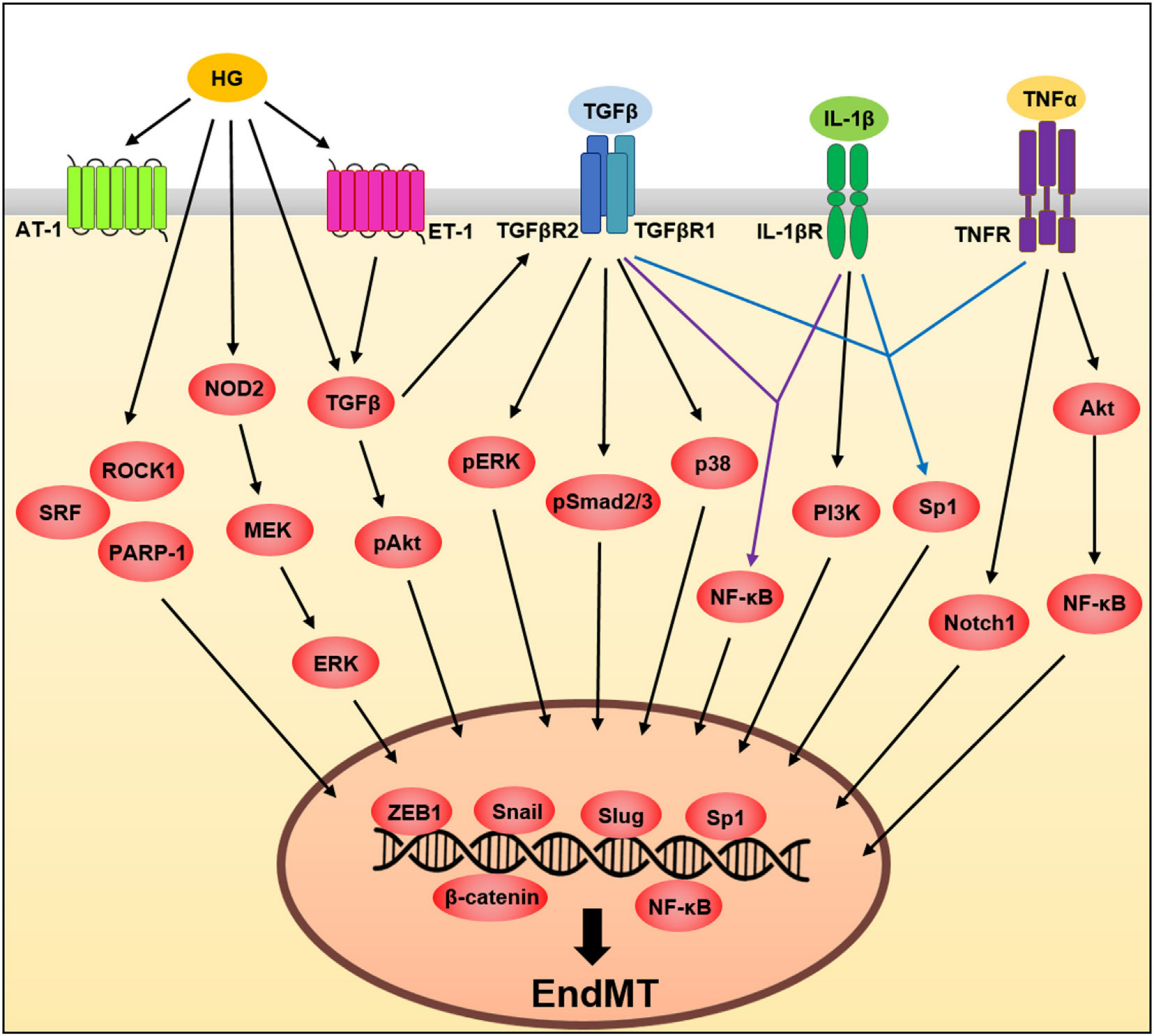
A schematic illustration of the signaling pathways governing endothelial to mesenchymal transition (EndMT). Tumor necrosis factor-α (TNF-α), transforming growth factor beta (TGFβ), interleukin (IL)-1β, and high glucose influences EndMT by regulating signaling pathways. These pathways converge and induce the expression of transcription factors involving Slug, Snail and zinc finger E-box-binding homeobox 1 (ZEB1) (see text for details).

Tumor necrosis factor-α, a pro-inflammatory cytokine, plays an important role in the regulation of various cellular activities (38). In ECs, TNF-α responses are initiated by the binding of one of two receptors, TNF receptor type 1 and TNF receptor type 2, allowing these receptors to activate transcription factors, such as NF-κB, which leads to the induction of transcription of multiple genes such as vascular cell adhesion molecule 1 (*VCAM-1*) and intercellular adhesion molecule 1 (31, 39–42). TNF-α also induces EndMT through activation of multiple signaling pathways in various ECs types (39, 40, 43, 44). However, future studies will be needed to clarify what type of TNF-α receptor is involved in EndMT. In lymphatic endothelium, TNF-α-induced EndMT occurs through inhibition of vascular endothelial (VE)-cadherin expression while increasing the expression of β-catenin, N-cadherin, and ZEB1, key molecules involved in the EndMT processes (44). A previous study (40) showed that TNF-α drives EndMT through Akt/NF-κB activity in both embryonic and adult-valve endothelium, finding that EndMT-related protein expression involving α-SMA and snail was significantly upregulated, whereas VE-cadherin was significantly downregulated in response to TNF-α in porcine aortic valve ECs (PAVECs), but not porcine aortic ECs (40); suggesting the importance of determining the molecular mechanism of EndMT in the context of endothelial heterogeneity during inflammation. The same group also demonstrated heterogeneous susceptibility to EndMT in PAVECs in response to TNF-α. Under TNF-α stimulation, non-transforming cells that maintain endothelial-cell marker expression and transforming cells that acquire mesenchymal-marker expression were isolated using membrane-based three-dimensional culture systems. Transforming cells decreased endothelial marker expression, such as VE-cadherin and endothelial nitric oxide synthase and acquired mesenchymal markers, such as α-SMA, Notch1, MMP-9, BMP-4, and TGFβ in PAVECs (Figure 2) (39).

Interleukin-1β is a proinflammatory cytokine (45) involved in endothelial dysfunction (46) and a key inducer of EndMT. IL-1β-induced phenotypic changes in ECs were first demonstrated in IL-1β-treated human dermal microvascular ECs undergoing morphological changes and cytoskeletal reorganization, in addition to decreased expression of typical endothelial markers, such as von Willebrand Factor (vWF) and CD31 (47). In addition, long-term exposure of human dermal microvascular ECs to IL-1β induces the expression of mesenchymal markers such as α-SMA, type I collagen, and calponin and inhibits the expression of vWF (48). Maleszewska et al. (49) reported that the molecular mechanism underlying IL-1β-induced EndMT involves increased expression of SM22α, which is encoded by *TAGLN*. Their results demonstrated the epigenetic regulation of *TAGLN via* EZH2, which acts as a key negative regulator in IL-1β-induced EndMT (49). Moreover, in corneal ECs, IL-1β induced an EndMT phenotype by increasing fibroblast growth factor (FGF) expression through the PI3K-signaling pathway (50, 51) in accordance with changes in the actin cytoskeleton and cellular morphology (Figure 2). The most recent study has shown that the NLRP3 inflammasome, closely associated with mature IL-1β secretion, is involved in mechanical stretch-induced EndMT in lung fibrosis and NLRP3 inactivation could inhibit EndMT, suggesting novel therapeutic options against mechanical ventilation-induced pulmonary fibrosis (52).

Several studies reported that a combination of cytokines, including TNF-α, IL-1β, and TGFβ, is more powerful than a single cytokine at inducing EndMT. The combination of TGFβ1, IL-1β, and TNF-α induces EndMT *via* the Sp1 transcription factor, which is a key transcriptional regulator of EndMT-related genes in human intestinal microvascular endothelial cells (31). TGFβ2 and IL-1β synergistically induce EndMT through increased expression of mesenchymal markers while decreasing the expression of endothelial markers in human esophageal microvascular endothelial cells and human umbilical vein ECs (HUVECs) (53, 54). The combination of TNF-α, IL-1β, and TGFβ1 also induces EndMT in pulmonary artery ECs, with EndMT cells exhibiting morphological changes, as well as changes in endothelial and mesenchymal markers (7).

Emerging evidence has shown that endothelial dysfunction induced by metabolic disorders such as obesity, hyperglycemia, and dyslipidemia is critically associated with induction of EndMT. Several studies have demonstrated that high glucose induces EndMT, which leads to increased expression of mesenchymal markers, such as α-SMA, FSP-1, type I collagen, fibronectin, vimentin, and MMP-2 along with decreased expression of endothelial markers CD31 and VE-cadherin in various EC types (55–57). It was shown that high-glucose-induced EndMT occurs through positive regulators, such as Smad2/3, Snail, Rho-associated kinase 1 (ROCK1), serum response factor (SRF), nucleotide-binding oligomerization domain-containing protein 2 (NOD2), and ERK in glomerular ECs (57–60). In human aortic ECs (HAECs) and HUVECs, high glucose also induces EndMT through positive regulators, such as angiotensin II, poly (ADP-ribose) polymerase 1 (PARP-1), endothelin 1 (ET-1), Smad, Akt, p38, and ERK, contributing to diabetic cardiomyopathy (55, 56, 61–63). It has also been shown that oxidized low-density lipoprotein (ox-LDL) accelerates radiation-induced EndMT in HAECs and contributes to radiation-induced atherosclerosis (64), whereas high-density lipoprotein (HDL) inhibits TGFβ1-induced EndMT in HAECs suggesting anti-fibrotic effects of HDL (Figure 2) (65).

Although much attention has recently been directed to EndMT because of its importance in many diseases, most studies have been limited to the identification of endothelial and mesenchymal markers in response to inducers of EndMT. Therefore, elucidation of the potential molecular mechanisms regulating pathological EndMT induced by inflammatory stimuli will be important in the future. Table 1 shows a summary of the main studies exploring EndMT under specific inflammatory stimuli and metabolic dysfunction, including EndMT mediators, endothelial and mesenchymal markers, and EC types.

**Table 1:** Summary of the key studies exploring EndMT under specific inflammatory stimuli and metabolic dysfunction.

Stimuli	Endothelial markers	Mesenchymal markers	Endothelial cell types	Positive regulator of EndMT	Reference
TNF-α	VE-cadherin	N-cadherin	LEC	ZEB1 and β-catenin	(44)
TNF-α	VE-cadherin, CD31, eNOS	α-SMA and MMP-9	PAVEC and eQEE	Akt/NF-κB, Snail, Slug, TGFβ, Notch1, and BMP-4	(39, 40)
IL-1β	vWF	α-SMA, collagen I, and calponin	HDMEC	Non determined	(47, 48)
IL-1β	Non determined	SM22α	HUVEC	pSmad2 and TGFβ2	(49)
IL-1β	Non determined	Cell shape change and actin cytoskeleton	CECs	PI3K	(50, 51)
TNF-α, IL-1β and TGFβ1	VE-cadherin, CD31, and vWF	α-SMA, FSP-1, vimentin, N-cadherin, and fibronectin	HIMEC	Sp1	(31)
TGFβ2 and IL-1β	CD31, vWF, and VE-cadherin	SM22α, FSP-1, collagen 1 A2, vimentin, and α-SMA	HEMEC	Snail	(53)
TGFβ2 and IL-1β	eNOS and vWF	SM22α, calponin	HUVEC	NF-κB	(54)
TNF-α, IL-1β and TGFβ1	vWF, CD31, VE-cadherin, and Occludin	Calponin, α-SMA, and collagen I	PAECs	Non determined	(7)
High glucose	CD31 and VE-cadherin	α-SMA, α-SMA, FSP-1, and fibronectin	GEnC	TGFβ, pSmad2/3, Snail, ROCK1, NOD1, MEK/ERK, SRF, and Snail	(57–60)
High glucose	CD31 and VE-cadherin	α-SMA, FSP-1, collagen I, collagen III, and MMP-2/9	HAEC	Angiotensin II, Snail, and PARP-1	(55, 61)
High glucose	VE-cadherin and CD31	α-SMA, collagen I, FSP-1, vimentin, and MMP-2/9	HUVEC	TGFβ1, ERK, pSmad2/3, and MAPK (p38 and ERK)	(56, 63)
High glucose	VE-cadherin	FSP-1 and collagen I	HUVEC and HAEC	ET-1, TGFβ1, pSmad3, pAKT, and Snail	(62)
ox-LDL + Radiation	VE-cadherin and CD31	α-SMA, FSP-1, and vimentin	HAEC	Non determined	(64)

*TNF-α, tumor necrosis factor-α; IL-1β, interleukin-1β; TGFβ, transforming growth factor-β; VE-cadherin, vascular endothelial cadherin; eNOS, endothelial nitric oxide synthase; vWF, von Willebrand Factor; α-SMA, α-smooth muscle actin; SM22α, smooth muscle protein 22-α; FSP-1, fibroblast-specific protein 1; LEC, lymphatic endothelial cell; PAVEC, porcine aortic valve endothelial cell; eQEE, embryonic quail endocardial explant; HDMEC, human epithelioid dermal microvascular endothelial cell; HUVEC, human umbilical vein endothelial cell; CEC, corneal endothelial cell; HIMEC, human intestinal microvascular endothelial cell; HEMEC, human esophageal microvascular endothelial cell; PAEC, pulmonary artery endothelial cell; ZEB1, zinc finger E-box-binding homeobox 1; NF-κB, nuclear factor kappa B; BMP-4, bone morphogenetic protein 4; EZH2, enhancer of zeste homolog 2; FGF-2, fibroblast growth factor 2; PI3K, phosphatidylinositol 3-kinase. GEnC, glomerular endothelial cell; ROCK1, Rho-associated kinase 1; HAEC, human aortic endothelial cell; GLP-1, glucagon-like peptide-1; PARP-1, Poly (ADP-ribose) polymerase 1; SRF, Serum response factor; ET-1, endothelin-1; ox-LDL, oxidized low-density lipoprotein; NOD2, Nucleotide-binding oligomerization domain-containing protein 2*.

## EC Heterogeneity During Inflammation

Endothelial cells line the inner wall of blood vessels and exhibit diverse subtypes (2, 8, 33). Different ECs have different structural and functional characteristics based on their exposure to distinct microenvironments (2, 8, 9, 33, 66). They are activated in response to inflammatory stimuli, with this activation resulting in the expression of adhesion molecules necessary for leukocyte binding (33).

Many studies showed that each EC subtype responds differently to different inflammatory stimuli *in vitro* (33). Viemann et al. (67) suggested that genes differentially regulated upon TNF-α stimulation between human microvascular ECs and HUVECs exhibit functional differences, and that genes whose expression was altered only in the human microvascular ECs group were associated with signaling and transcription factors, apoptosis, cell proliferation, immune response, and cell structure. However, genes showing altered expression only in HUVECs were associated with chemokines, cytokines, cell-surface molecules, and signaling and transcription factors (67). VCAM-1 expression was only increased in response to TNF-α in HUVECs and glomerular ECs, but not in dermal microvascular ECs (68). In addition, Scott et al. (69) reported changes in heterogeneous gene expression in response to TNF-α, lipopolysaccharide (LPS), and IL-1β in HUVECs, human pulmonary microvascular ECs, HAECs, carotid artery ECs, coronary artery ECs, subclavian artery ECs, and brachiocephalic artery ECs. TNF-α, and IL-1β-stimulated organ-specific endothelial heterogeneity has also been reported (70).

These findings suggest that each EC subtype might respond differently to different inflammatory stimuli in the context of EndMT. Indeed, Pinto et al. (71) compared responses to TGFβ1 or TGFβ2 between human coronary artery ECs and microvascular pulmonary artery ECs, finding that in human coronary artery ECs, both TGFβ1 and TGFβ2 upregulated the expression of the mesenchymal markers α-SMA and SM22α, but only TGFβ1 had an effect on α-SMA expression in human pulmonary microvascular ECs. In addition, TNF-α increased the expression of the mesenchymal markers in PAVECs, although this response was not observed in porcine aortic ECs (40).

Importantly, other inflammation-associated endothelial activators, such as shear stress or protein kinase C (PKC), have also been studied in this context (33). Methe et al. (72) showed that venous and coronary artery specific flows differentially regulate the expression of endothelial adhesion molecules as well as KLF2/KLF4 transcription factors in human saphenous vein ECs and human coronary artery ECs. Two ECs have also been reported to show heterogeneity in adhesion molecule expression in response to PKC. Here, PKC activation induces E-selectin and VCAM-1 expression in HUVECs, but not in human dermal microvascular ECs (73).

Differences in the behavior of various EC subtypes in response to inflammatory stimuli have also been reported *in vivo* (33). Tamaru et al. (74) showed that induction of adhesion molecule expression in response to IL-1β stimulation is both tissue- and cell-type specific. However, no changes were observed in VCAM-1 expression in brain and liver microvascular ECs in response to LPS stimulation (75, 76). Furthermore, van Meurs et al. (77) showed that E-selectin and VCAM expression in human glomerular ECs differs from that in other ECs. In CD31-deficient mice, apoptosis of peritubular-capillary ECs occurs upon LPS administration, although other microvessel ECs were unaffected (78). Given the extent of endothelial heterogeneity found both *in vitro* and *in vivo*, studying the molecular mechanisms and functions associated with the EndMT process in inflammation in the context of endothelial heterogeneity will eventually enable us to better understand vascular diseases and develop more sophisticated and effective therapeutic drugs.

## Targeting ENDMT for Therapeutic and Clinical Applications in Vascular Diseases

Endothelial to mesenchymal transition is recognized to not only occur during development but also it is now clear that EndMT underlies pathological processes associated with multiple diseases (6, 26, 79, 80). EndMT is also controlled by a variety of stimuli, including inflammation, growth factors, and hypoxia (81–83). Particularly, inflammation-induced EndMT aggravates inflammation and destroys vascular homeostasis, leading to pathogenesis of several diseases, such as cardiac fibrosis, PAH, and atherosclerosis (31, 84, 85). Given the involvement of EndMT in multiple inflammatory diseases, preventing EndMT may represent a useful approach to treat inflammatory diseases.

Several factors have been identified as the negative regulators of EndMT signaling pathways (86–88). Vascular endothelial growth factor-A reverses TGFβ2-induced EndMT (89), and HDL and the extracellular-matrix protein fibulin 1 and kallistatin also exert inhibitory effects on TGFβ-induced EndMT (65, 90, 91). Furthermore, the most common aldosterone receptor antagonist, spironolactone has a protective role against TGFβ-induced EndMT in HUVECs (92) and, rapamycin suppresses mechanistic target of mTOR signaling, leading to the inhibition of EndMT (93). Although emerging studies report multiple EndMT mediators that play critical roles in EndMT induction, the targeting of EndMT mediators requires careful evaluation due to the modulation of EndMT exhibiting differential effects in different ECs based on endothelial heterogeneity. For example, IL-1β upregulates FGF2 expression through PI3K activation, which leads to EndMT of corneal ECs (51). Lee et al. (50) suggested that blocking the IL-1β and FGF2 pathways would prevent inflammation-induced EndMT in corneal ECs; however, FGF2 exerts an inhibitory effect on TGFβ-mediated EndMT *via* miR-20a in HUVECs (81, 94). Moreover, another study showed that FGF receptor-1 is a key inhibitor of TGFβ-driven EndMT in HUVECs (95), and the endogenous antifibrotic peptide N-acetyl-seryl-aspartyl-lysyl-proline restores FGF receptor levels and upregulates levels of the let-7, resulting in the inhibition of EndMT in human dermal microvascular ECs (96). Therefore, further studies are needed to completely elucidate the mechanisms associated with FGF2, as well as many mediators involved in EndMT (81).

In the context of diseases, studies on the inhibitory effect of EndMT have mainly focused on fibrosis. The common feature of many fibro-proliferative diseases is inflammation (97). Fibrosis results from chronic inflammation, possibly owing to infection, autoimmune reactions, or allergic reactions, which results in the release of inflammatory mediators, abnormal cell proliferation, and deposition of extracellular-matrix components (81, 82, 84, 97). BMP7 has been shown to exhibit anti-EndMT effects and reduce cardiac fibrosis; however, most other BMPs are positive regulators of EndMT (81, 85, 98, 99). Hepatocyte growth factor reduces cardiac fibrosis by suppressing TGFβ1-mediated EndMT (100). Cinacalcet, a calcimimetic agent, reduces serum levels of parathyroid hormone and suppresses EndMT, leading to attenuation of cardiac fibrosis (101). Similarly, losartan and irbesartan, two angiotensin II-receptor type 1 blockers, ameliorate cardiac fibrosis by inhibiting EndMT (61, 102). Scutellarin and relaxin are also EndMT inhibitors and prevent cardiac fibrosis by regulating Notch1 and Jagged-1 (86, 103). Furthermore, inhibition of CD45 protein tyrosine phosphatase leads to reduced EndMT in TGFβ1-treated mitral valve ECs (104). The anti-fibrotic effects of linagliptin, which blocks EndMT, have also been reported *in vitro* and in diabetic kidneys (105). Similarly, macitentan inhibits endothelin-1 or TGFβ1-induced EndMT in systemic sclerosis (106).

Cytokine-induced inflammation is widely considered a major cause of PAH development (107). Moreover, remodeling of the pulmonary artery under inflammatory conditions is a major feature of PAH (12–15). Recent evidence suggests that inflammation-induced EndMT is a key contributor to pathological pulmonary vascular remodeling associated with transition of ECs to α-SMA-expressing mesenchymal-like cells in obstructive vascular lesions of PAH (7, 29). Clinical data also indicate that the serum levels of IL-1, -6, -8, -10, and TNF-α are elevated in PAH patients (107). In this context, salvianolic acid A, a polyphenol compound, inhibits EndMT in PAH, thereby attenuating inflammation associated with monocrotaline-induced PAH (108). Another study showed that the delivery of BMP receptor-2 resulted in less right-ventricle hypertrophy, pulmonary vascular resistance, and improved cardiac function through attenuation of EndMT (109). Kang et al. (87) suggested that ponatinib, a multi-target tyrosine-kinase inhibitor, delays TGFβ1-mediated EndMT and has therapeutic potential for use in PAH therapy, where it could act by regulating Wnt signaling.

Atherosclerosis is a vascular disease mediated by a typical inflammatory response. Inflammatory stimuli continuously lead to calcified plaque formation (110). Atherosclerosis lesions mostly comprise EndMT-derived fibroblast-like cells, which are regulated by various EndMT mediators, such as snail, slug, and β-catenin (84, 111, 112). Other pathways might also lead to atherosclerosis by inducing EndMT *via* TGFβ, oxidative stress, hypoxia, Wnt/β-catenin signaling, and BMP signaling (81, 84, 113). These data suggest that EndMT is a major source of neointimal hyperplasia and plays a role in the progression of arteriosclerosis through inflammation. In particular, excessive BMP activity promotes the calcification of atherosclerotic lesions through EndMT and serine-protease inhibitors also reduce EndMT and vascular calcification (88, 114). Consistent with this report, vascular calcification was found to be reduced in response to a BMP inhibitor in matrix-gla-protein-deficient mice (115).

It has been shown that metabolic syndrome, which is associated with metabolic dysfunction such as obesity, hyperglycemia, insulin resistance, and dyslipidemia has a central role in the pathogenesis of cardiovascular disease, diabetes mellitus type 2, and tissue remodeling (116). Abnormalities associated with chronic inflammation are key risk factors of metabolic dysfunction, leading to the endothelial dysfunction that is critically involved in the development of such diseases (64, 116–119). In the context of metabolic syndrome, studies on EndMT induced by metabolic dysfunction have mainly focused on diabetic nephropathy and cardiomyopathy. Several studies have shown that EndMT contributes to diabetic nephropathy, while inhibition of EndMT by lovastatin, fasudil, and CCG-1423 could ameliorate diabetic nephropathy in streptozotocin (STZ)-induced diabetic animal models (57–59). In addition, inhibition of EndMT by Irbesartan, glucagon-like peptide-1 analog, ET-1 inhibition and low-dose irisin could prevent diabetic cardiomyopathy in diabetic animal models (55, 61–63).

Taken together, the currently available data indicate that EndMT plays a key role in various fibrosis-related and cardiovascular diseases (Figure 1; Table 2). Considering the large number of studies that suggest targeting EndMT as a novel therapeutic approach for many diseases, clarifying the underlying signaling mechanisms associated with EndMT and establishing strategies to regulate EndMT are urgently needed.

**Table 2:** Summary of the key studies exploring endothelial to mesenchymal transition (EndMT) as a therapeutic target in various diseases.

Model of Study	Negative regulator of EndMT	Clinical relevance	Reference
Isoproterenol-induced myocardial fibrosis rat model	Relaxin	Cardiac fibrosis	(86)
Bleomycin-induced PAH model	Ponatinib (multi-targeted tyrosine-kinase inhibitor)	Pulmonary arterial hypertension (PAH)	(87)
TGFβ1-induced EndMT	HDL	Non-determined	(65)
TGFβ-induced EndMT	Spironolactone (aldosterone receptor antagonist)	Non-determined	(92)
Mouse models of pressure overload and chronic allograft rejection	BMP-7	Cardiac fibrosis	(85)
Heterotopic heart transplantation model	BMP-7	Endocardial fibroelastosis	(99)
Pressure-overload mouse model	HGF	Cardiac fibrosis	(100)
Rat model of uremia and secondary hyperparathyroidism	Cinacalcet (calcimimetic agent)	Cardiac fibrosis	(101)
TGFβ1-induced EndMT	Losartan (angiotensin II receptor type 1 blocker)	Non determined	(102)
Isoproterenol -induced myocardial fibrosis rat model	Scutellarin	Cardiac fibrosis	(103)
Ovine inferior myocardial infarction model	CD45-selective PTPase inhibitor	Myocardial infarction	(104)
STZ-induced diabetic mice	Linagliptin (DPP-4 inhibitor)	Diabetic kidney fibrosis	(105)
TGFβ and ET-1-induced EndMT	Macitentan (ET-1 receptor antagonist)	Systemic sclerosis	(106)
MCT-induced PAH model	Salvianolic acid A	Pulmonary arterial hypertension	(108)
Hypoxia, MCT-induced PAH model	Delivery of *BMPR2*	Pulmonary arterial hypertension	(109)
STZ-induced diabetic rats	Lovastatin	Diabetic nephropathy	(58)
*db/db* diabetic mice	Fasudil (ROCK1 inhibitor)	Diabetic nephropathy	(59)
STZ-induced SHR diabetic rats	Irbesartan (angiotensin II receptor type 1 blocker)	Diabetic cardiomyopathy	(61)
STZ-induced diabetic mice	GLP-1 analog	Diabetic cardiomyopathy	(55)
STZ-induced diabetic rats	CCG-1423 (SRF inhibitor)	Diabetic nephropathy	(57)
STZ-induced diabetic ET-1^f/f^; Tie2-Cre(+) mice	ET-1 silencing	Diabetic cardiomyopathy	(62)
STZ-induced diabetic mice	Low-dose irisin	Diabetic cardiomyopathy	(63)
TGFβ1-induced EndMT	HDL	Non determined	(65)

*TNF-α, tumor necrosis factor-α; IL-1β, interleukin-1β; TGFβ, transforming growth factor-β; VE-cadherin, vascular endothelial cadherin; eNOS, endothelial nitric oxide synthase; vWF, von Willebrand factor; α-SMA, α-smooth muscle actin; SM22α, smooth muscle protein 22-α; FSP-1, fibroblast-specific protein 1; LEC, lymphatic endothelial cell; PAVEC, porcine aortic valve endothelial cell; eQEE, embryonic quail endocardial explant; HDMEC, human epithelioid dermal microvascular endothelial cell; HUVEC, human umbilical vein endothelial cell; CEC, corneal endothelial cell; HIMEC, human intestinal microvascular endothelial cell; HEMEC, human esophageal microvascular endothelial cell; PAEC, pulmonary artery endothelial cell; ZEB1, zinc finger E-box-binding homeobox 1; NF-κB, nuclear factor kappa B; BMP-4, bone morphogenetic protein 4; EZH2, enhancer of zeste homolog 2; FGF-2, fibroblast growth factor 2; PI3K, phosphatidylinositol 3-kinase. STZ, streptozotocin; SHR, spontaneously hypertensive rats; HDL, High-Density Lipoproteins; HGF, hepatocyte growth factor; MCT, monocrotaline*.

## Conclusion

Endothelial to mesenchymal transition plays an important role not only during the development process but also in adults under physiological and pathological conditions. A central role for EndMT emerges from the complex network of interactions that underlie inflammation-induced endothelial dysfunction. There is accumulating evidence indicating that EndMT is a key feature in inflammation-related endothelial dysfunction. It is through this phenotypic switch that EndMT causes diverse vascular diseases, such as atherosclerosis, PAH, and fibrosis. Therefore, the modulation of EndMT might yield new therapeutic strategies for the treatment of diverse diseases. Although our current understanding of the molecular mechanisms underlying EndMT in the context of inflammation is advancing, further studies are needed in the future to completely understand the molecular mechanism associated with EndMT in inflammation-related diseases. Given that heterogeneity is apparent in ECs of different organs in response to different inflammatory stimuli, it will also be important to determine the molecular mechanisms associated with EndMT in the context of endothelial heterogeneity during inflammation in future studies. In conclusion, the study of EndMT will provide valuable insights into the molecular mechanisms leading to various human diseases and will help develop more sophisticated and effective therapeutic drugs for patients suffering from these diseases.

## Author Contributions

JK, JC, and AL wrote the manuscript. JK, WC, and MS-L were critically involved in the design of the work and the discussion of the content. All the authors approved the final manuscript.

## Conflict of Interest Statement

The authors declare that this research was conducted in the absence of any commercial or financial relationships that could be construed as a potential conflict of interest.

## References

[1] Sena CM Pereira AM Seica R. Endothelial dysfunction – a major mediator of diabetic vascular disease. Biochim Biophys Acta (2013) 1832(12):2216–31. 10.1016/j.bbadis.2013.08.006 23994612

[2] Aird WC. Endothelial cell heterogeneity. Cold Spring Harb Perspect Med (2012) 2(1):a006429. 10.1101/cshperspect.a006429 22315715 PMC3253027

[3] Gimbrone MA Jr Garcia-Cardena G. Endothelial cell dysfunction and the pathobiology of atherosclerosis. Circ Res (2016) 118(4):620–36. 10.1161/CIRCRESAHA.115.306301 26892962 PMC4762052

[4] Vanhoutte PM Shimokawa H Feletou M Tang EH. Endothelial dysfunction and vascular disease – a 30th anniversary update. Acta Physiol (Oxf) (2017) 219(1):22–96. 10.1111/apha.12646 26706498

[5] Juni RP Duckers HJ Vanhoutte PM Virmani R Moens AL. Oxidative stress and pathological changes after coronary artery interventions. J Am Coll Cardiol (2013) 61(14):1471–81. 10.1016/j.jacc.2012.11.068 23500310

[6] Perez L Munoz-Durango N Riedel CA Echeverria C Kalergis AM Cabello-Verrugio C Endothelial-to-mesenchymal transition: cytokine-mediated pathways that determine endothelial fibrosis under inflammatory conditions. Cytokine Growth Factor Rev (2017) 33:41–54. 10.1016/j.cytogfr.2016.09.002 27692608

[7] Good RB Gilbane AJ Trinder SL Denton CP Coghlan G Abraham DJ Endothelial to mesenchymal transition contributes to endothelial dysfunction in pulmonary arterial hypertension. Am J Pathol (2015) 185(7):1850–8. 10.1016/j.ajpath.2015.03.019 25956031

[8] Kim JD Lee HW Jin SW. Diversity is in my veins: role of bone morphogenetic protein signaling during venous morphogenesis in *zebrafish* illustrates the heterogeneity within endothelial cells. Arterioscler Thromb Vasc Biol (2014) 34(9):1838–45. 10.1161/ATVBAHA.114.303219 25060789 PMC4140958

[9] Aird WC. Phenotypic heterogeneity of the endothelium: II. representative vascular beds. Circ Res (2007) 100(2):174–90. 10.1161/01.RES.0000255690.03436.ae 17272819

[10] Rajendran P Rengarajan T Thangavel J Nishigaki Y Sakthisekaran D Sethi G The vascular endothelium and human diseases. Int J Biol Sci (2013) 9(10):1057–69. 10.7150/ijbs.7502 24250251 PMC3831119

[11] Lee A Papangeli I Park Y Jeong HN Choi J Kang H A PPARgamma-dependent miR-424/503-CD40 axis regulates inflammation mediated angiogenesis. Sci Rep (2017) 7(1):2528 10.1038/s41598-017-02852-4 28566713 PMC5451412

[12] Lee A McLean D Choi J Kang H Chang W Kim J. Therapeutic implications of microRNAs in pulmonary arterial hypertension. BMB Rep (2014) 47(6):311–7. 10.5483/BMBRep.2014.47.6.085 24755557 PMC4163875

[13] Kim JD Lee A Choi J Park Y Kang H Chang W Epigenetic modulation as a therapeutic approach for pulmonary arterial hypertension. Exp Mol Med (2015) 47:e175. 10.1038/emm.2015.45 26228095 PMC4525299

[14] Kim J Kang Y Kojima Y Lighthouse JK Hu X Aldred MA An endothelial apelin-FGF link mediated by miR-424 and miR-503 is disrupted in pulmonary arterial hypertension. Nat Med (2013) 19(1):74–82. 10.1038/nm.3040 23263626 PMC3540168

[15] Kim J Hwangbo C Hu X Kang Y Papangeli I Mehrotra D Restoration of impaired endothelial myocyte enhancer factor 2 function rescues pulmonary arterial hypertension. Circulation (2015) 131(2):190–9. 10.1161/CIRCULATIONAHA.114.013339 25336633 PMC4293354

[16] Galle J Quaschning T Seibold S Wanner C. Endothelial dysfunction and inflammation: What is the link? Kidney Int (2003) 63:S45–9. 10.1046/j.1523-1755.63.s84.12.x 12694307

[17] Zhang C. The role of inflammatory cytokines in endothelial dysfunction. Basic Res Cardiol (2008) 103(5):398–406. 10.1007/s00395-008-0733-0 18600364 PMC2705866

[18] Chen Y Pitzer AL Li X Li PL Wang L Zhang Y. Instigation of endothelial Nlrp3 inflammasome by adipokine visfatin promotes inter-endothelial junction disruption: role of HMGB1. J Cell Mol Med (2015) 19(12):2715–27. 10.1111/jcmm.12657 26293846 PMC4687695

[19] Vandanmagsar B Youm YH Ravussin A Galgani JE Stadler K Mynatt RL The NLRP3 inflammasome instigates obesity-induced inflammation and insulin resistance. Nat Med (2011) 17(2):179–88. 10.1038/nm.2279 21217695 PMC3076025

[20] Varghese GP Folkersen L Strawbridge RJ Halvorsen B Yndestad A Ranheim T NLRP3 inflammasome expression and activation in human atherosclerosis. J Am Heart Assoc (2016) 5(5):e003031 10.1161/JAHA.115.003031 27207962 PMC4889178

[21] Franchi L Munoz-Planillo R Nunez G. Sensing and reacting to microbes through the inflammasomes. Nat Immunol (2012) 13(4):325–32. 10.1038/ni.2231 22430785 PMC3449002

[22] Chen Z Martin M Li Z Shyy JY. Endothelial dysfunction: the role of sterol regulatory element-binding protein-induced NOD-like receptor family pyrin domain-containing protein 3 inflammasome in atherosclerosis. Curr Opin Lipidol (2014) 25(5):339–49. 10.1097/MOL.0000000000000107 25188917 PMC4339278

[23] Cerretti DP Kozlosky CJ Mosley B Nelson N Van Ness K Greenstreet TA Molecular cloning of the interleukin-1 beta converting enzyme. Science (1992) 256(5053):97–100. 10.1126/science.1373520 1373520

[24] Thornberry NA Bull HG Calaycay JR Chapman KT Howard AD Kostura MJ A novel heterodimeric cysteine protease is required for interleukin-1 beta processing in monocytes. Nature (1992) 356(6372):768–74. 10.1038/356768a0 1574116

[25] He Y Zeng MY Yang DH Metro B Nunez G. NEK7 is an essential mediator of NLRP3 activation downstream of potassium efflux. Nature (2016) 530(7590):354–+. 10.1038/nature16959 26814970 PMC4810788

[26] Chen PY Simons M When endothelial cells go rogue. EMBO Mol Med (2016) 8(1):1–2. 10.15252/emmm.201505943 26613939 PMC4718160

[27] Ghosh AK Quaggin SE Vaughan DE Molecular basis of organ fibrosis: potential therapeutic approaches. Exp Biol Med (Maywood) (2013) 238(5):461–81. 10.1177/1535370213489441 23856899

[28] Potenta S Zeisberg E Kalluri R. The role of endothelial-to-mesenchymal transition in cancer progression. Br J Cancer (2008) 99(9):1375–9. 10.1038/sj.bjc.6604662 18797460 PMC2579683

[29] Arciniegas E Frid MG Douglas IS Stenmark KR. Perspectives on endothelial-to-mesenchymal transition: potential contribution to vascular remodeling in chronic pulmonary hypertension. Am J Physiol Lung Cell Mol Physiol (2007) 293(1):L1–8. 10.1152/ajplung.00378.2006 17384082

[30] Sanchez-Duffhues G Orlova V Ten Dijke P. In brief: endothelial-to-mesenchymal transition. J Pathol (2016) 238(3):378–80. 10.1002/path.4653 26446982

[31] Rieder F Kessler SP West GA Bhilocha S de la Motte C Sadler TM Inflammation-induced endothelial-to-mesenchymal transition: a novel mechanism of intestinal fibrosis. Am J Pathol (2011) 179(5):2660–73. 10.1016/j.ajpath.2011.07.042 21945322 PMC3204019

[32] Welch-Reardon KM Wu N Hughes CC. A role for partial endothelial-mesenchymal transitions in angiogenesis? Arterioscler Thromb Vasc Biol (2015) 35(2):303–8. 10.1161/ATVBAHA.114.303220 25425619 PMC4911209

[33] Dauphinee SM Karsan A Endothelial Dysfunction and Inflammation. Basel: Birkhäuser (2010). xii,234 p.

[34] Gori T Dragoni S Di Stolfo G Forconi S. Endothelium and haemorheology. Ann Ist Super Sanita (2007) 43(2):124–9. 17634660

[35] Dejana E Hirschi KK Simons M The molecular basis of endothelial cell plasticity. Nat Commun (2017) 8:14361 10.1038/ncomms14361 28181491 PMC5309780

[36] Gonzalez DM Medici D. Signaling mechanisms of the epithelial-mesenchymal transition. Sci Signal (2014) 7(344):re8. 10.1126/scisignal.2005189 25249658 PMC4372086

[37] Yu QC Song W Wang D Zeng YA. Identification of blood vascular endothelial stem cells by the expression of protein C receptor. Cell Res (2016) 26(10):1079–98. 10.1038/cr.2016.85 27364685 PMC5113308

[38] MacEwan DJ TNF ligands and receptors – a matter of life and death. Br J Pharmacol (2002) 135(4):855–75. 10.1038/sj.bjp.0704549 11861313 PMC1573213

[39] Farrar EJ Butcher JT Heterogeneous susceptibility of valve endothelial cells to mesenchymal transformation in response to TNFalpha. Ann Biomed Eng (2014) 42(1):149–61. 10.1007/s10439-013-0894-3 23982279 PMC3905205

[40] Mahler GJ Farrar EJ Butcher JT. Inflammatory cytokines promote mesenchymal transformation in embryonic and adult valve endothelial cells. Arterioscler Thromb Vasc Biol (2013) 33(1):121–30. 10.1161/ATVBAHA.112.300504 23104848 PMC3694265

[41] Al-Soudi A Kaaij MH Tas SW. Endothelial cells: from innocent bystanders to active participants in immune responses. Autoimmun Rev (2017) 16(9):951–62. 10.1016/j.autrev.2017.07.008 28698091

[42] Modur V Zimmerman GA Prescott SM McIntyre TM. Endothelial cell inflammatory responses to tumor necrosis factor alpha. ceramide-dependent and -independent mitogen-activated protein kinase cascades. J Biol Chem (1996) 271(22):13094–102. 10.1074/jbc.271.22.13094 8662702

[43] Wu KQ Muratore CS So EY Sun C Dubielecka PM Reginato AM M1 macrophage-induced endothelial-to-mesenchymal transition promotes infantile hemangioma regression. Am J Pathol (2017) 187(9):2102–11. 10.1016/j.ajpath.2017.05.014 28710904 PMC5809337

[44] Chakraborty S Zawieja DC Davis MJ Muthuchamy M. MicroRNA signature of inflamed lymphatic endothelium and role of miR-9 in lymphangiogenesis and inflammation. Am J Physiol Cell Physiol (2015) 309(10):C680–92. 10.1152/ajpcell.00122.2015 26354749 PMC4652079

[45] Dinarello CA. Proinflammatory cytokines. Chest (2000) 118(2):503–8. 10.1378/chest.118.2.503 10936147

[46] Bhagat K Vallance P. Inflammatory cytokines impair endothelium-dependent dilatation in human veins in vivo. Circulation (1997) 96(9):3042–7. 10.1161/01.CIR.96.9.3042 9386173

[47] Romero LI Zhang DN Herron GS Karasek MA. Interleukin-1 induces major phenotypic changes in human skin microvascular endothelial cells. J Cell Physiol (1997) 173(1):84–92. 10.1002/(SICI)1097-652(199710)173:1<84:AID-JCP10>3.0.CO;2-N 9326452

[48] Chaudhuri V Zhou L Karasek M. Inflammatory cytokines induce the transformation of human dermal microvascular endothelial cells into myofibroblasts: a potential role in skin fibrogenesis. J Cutan Pathol (2007) 34(2):146–53. 10.1111/j.1600-0560.2006.00584.x 17244026

[49] Maleszewska M Gjaltema RA Krenning G Harmsen MC Enhancer of zeste homolog-2 (EZH2) methyltransferase regulates transgelin/smooth muscle-22alpha expression in endothelial cells in response to interleukin-1beta and transforming growth factor-beta2. Cell Signal (2015) 27(8):1589–96. 10.1016/j.cellsig.2015.04.008 25917318

[50] Lee JG Ko MK Kay EP Endothelial mesenchymal transformation mediated by IL-1beta-induced FGF-2 in corneal endothelial cells. Exp Eye Res (2012) 95(1):35–9. 10.1016/j.exer.2011.08.003 21855543

[51] Lee HT Lee JG Na M Kay EP. FGF-2 induced by interleukin-1 beta through the action of phosphatidylinositol 3-kinase mediates endothelial mesenchymal transformation in corneal endothelial cells. J Biol Chem (2004) 279(31):32325–32. 10.1074/jbc.M405208200 15173165

[52] Lv Z Wang Y Liu YJ Mao YF Dong WW Ding ZN NLRP3 inflammasome activation contributes to mechanical stretch-induced endothelial-mesenchymal transition and pulmonary fibrosis. Crit Care Med (2018) 46(1):e49–58. 10.1097/CCM.0000000000002799 29088003

[53] Nie L Lyros O Medda R Jovanovic N Schmidt JL Otterson MF Endothelial-mesenchymal transition in normal human esophageal endothelial cells cocultured with esophageal adenocarcinoma cells: role of IL-1beta and TGF-beta2. Am J Physiol Cell Physiol (2014) 307(9):C859–77. 10.1152/ajpcell.00081.2014 25163519 PMC4216936

[54] Maleszewska M Moonen JR Huijkman N van de Sluis B Krenning G Harmsen MC IL-1beta and TGFbeta2 synergistically induce endothelial to mesenchymal transition in an NFkappaB-dependent manner. Immunobiology (2013) 218(4):443–54. 10.1016/j.imbio.2012.05.026 22739237

[55] Yan F Zhang GH Feng M Zhang W Zhang JN Dong WQ Glucagon-Like peptide 1 protects against hyperglycemic-induced endothelial-to-mesenchymal transition and improves myocardial dysfunction by suppressing poly(ADP-Ribose) polymerase 1 activity. Mol Med (2015) 21:15–25. 10.2119/molmed.2014.00259 25715248 PMC4461581

[56] Yu CH Suriguga Gong M Liu WJ Cui NX Wang Y High glucose induced endothelial to mesenchymal transition in human umbilical vein endothelial cell. Exp Mol Pathol (2017) 102(3):377–83. 10.1016/j.yexmp.2017.03.007 28347704

[57] Zhao L Zhao J Wang X Chen Z Peng K Lu X Serum response factor induces endothelial-mesenchymal transition in glomerular endothelial cells to aggravate proteinuria in diabetic nephropathy. Physiol Genomics (2016) 48(10):711–8. 10.1152/physiolgenomics.00082.2016 27565710

[58] Ma Z Zhu L Liu Y Wang Z Yang Y Chen L Lovastatin alleviates endothelial-to-mesenchymal transition in glomeruli via suppression of oxidative stress and TGF-beta1 signaling. Front Pharmacol (2017) 8:473 10.3389/fphar.2017.00473 28769803 PMC5513942

[59] Peng H Li Y Wang C Zhang J Chen Y Chen W ROCK1 induces endothelial-to-mesenchymal transition in glomeruli to aggravate albuminuria in diabetic nephropathy. Sci Rep (2016) 6:20304. 10.1038/srep20304 26842599 PMC4740844

[60] Shang J Zhang Y Jiang Y Li Z Duan Y Wang L NOD2 promotes endothelial-to-mesenchymal transition of glomerular endothelial cells via MEK/ERK signaling pathway in diabetic nephropathy. Biochem Biophys Res Commun (2017) 484(2):435–41. 10.1016/j.bbrc.2017.01.155 28137583

[61] Tang RN Lv LL Zhang JD Dai HY Li Q Zheng M Effects of angiotensin II receptor blocker on myocardial endothelial-to-mesenchymal transition in diabetic rats. Int J Cardiol (2013) 162(2):92–9. 10.1016/j.ijcard.2011.06.052 21704391

[62] Widyantoro B Emoto N Nakayama K Anggrahini DW Adiarto S Iwasa N Endothelial cell-derived endothelin-1 promotes cardiac fibrosis in diabetic hearts through stimulation of endothelial-to-mesenchymal transition. Circulation (2010) 121(22):2407–18. 10.1161/CIRCULATIONAHA.110.938217 20497976

[63] Liu X Mujahid H Rong B Lu QH Zhang W Li P Irisin inhibits high glucose-induced endothelial-to-mesenchymal transition and exerts a dose-dependent bidirectional effect on diabetic cardiomyopathy. J Cell Mol Med (2017) 22(2):808–22. 10.1111/jcmm.13360 29063670 PMC5783871

[64] Kim M Choi SH Jin YB Lee HJ Ji YH Kim J The effect of oxidized low-density lipoprotein (ox-LDL) on radiation-induced endothelial-to-mesenchymal transition. Int J Radiat Biol (2013) 89(5):356–63. 10.3109/09553002.2013.763193 23289363

[65] Spillmann F Miteva K Pieske B Tschope C Van Linthout S. High-density lipoproteins reduce endothelial-to-mesenchymal transition. Arterioscler Thromb Vasc Biol (2015) 35(8):1774–7. 10.1161/ATVBAHA.115.305887 26088574

[66] Aird WC. Phenotypic heterogeneity of the endothelium I. Structure, function, and mechanisms. Circ Res (2007) 100(2):158–73. 10.1161/01.RES.0000255691.76142.4a 17272818

[67] Viemann D Goebeler M Schmid S Nordhues U Klimmek K Sorg C TNF induces distinct gene expression programs in microvascular and macrovascular human endothelial cells. J Leukoc Biol (2006) 80(1):174–85. 10.1189/jlb.0905530 16617158

[68] Murakami S Morioka T Nakagawa Y Suzuki Y Arakawa M Oite T. Expression of adhesion molecules by cultured human glomerular endothelial cells in response to cytokines: comparison to human umbilical vein and dermal microvascular endothelial cells. Microvasc Res (2001) 62(3):383–91. 10.1006/mvre.2001.2356 11678640

[69] Scott DW Vallejo MO Patel RP. Heterogenic endothelial responses to inflammation: role for differential N-glycosylation and vascular bed of origin. J Am Heart Assoc (2013) 2(4):e000263. 10.1161/JAHA.113.000263 23900214 PMC3828811

[70] Invernici G Ponti D Corsini E Cristini S Frigerio S Colombo A Human microvascular endothelial cells from different fetal organs demonstrate organ-specific CAM expression. Exp Cell Res (2005) 308(2):273–82. 10.1016/j.yexcr.2005.04.033 15936757

[71] Pinto MT Covas DT Kashima S Rodrigues CO. Endothelial mesenchymal transition: comparative analysis of different induction methods. Biol Proced Online (2016) 18:10. 10.1186/s12575-016-0040-3 27127420 PMC4848831

[72] Methe H Balcells M Alegret MD Santacana M Molins B Hamik A Vascular bed origin dictates flow pattern regulation of endothelial adhesion molecule expression. Am J Physiol Heart Circ Physiol (2007) 292(5):H2167–75. 10.1152/ajpheart.00403.2006 17209004

[73] Mason JC Yarwood H Sugars K Haskard DO. Human umbilical vein and dermal microvascular endothelial cells show heterogeneity in response to PKC activation. Am J Physiol (1997) 273(4 Pt 1):C1233–40. 10.1152/ajpcell.1997.273.4.C1233 9357767

[74] Tamaru M Tomura K Sakamoto S Tezuka K Tamatani T Narumi S. Interleukin-1beta induces tissue- and cell type-specific expression of adhesion molecules in vivo. Arterioscler Thromb Vasc Biol (1998) 18(8):1292–303. 10.1161/01.ATV.18.8.1292 9714137

[75] Yano K Okada Y Beldi G Shih SC Bodyak N Okada H Elevated levels of placental growth factor represent an adaptive host response in sepsis. J Exp Med (2008) 205(11):2623–31. 10.1084/jem.20080398 18852292 PMC2571936

[76] Shapiro NI Yano K Sorasaki M Fischer C Shih SC Aird WC. Skin biopsies demonstrate site-specific endothelial activation in mouse models of sepsis. J Vasc Res (2009) 46(5):495–502. 10.1159/000210662 19346756

[77] van Meurs M Wulfert FM Knol AJ De Haes A Houwertjes M Aarts LP Early organ-specific endothelial activation during hemorrhagic shock and resuscitation. Shock (2008) 29(2):291–9. 10.1097/SHK.0b013e318145a7c1 17704730

[78] Carrithers M Tandon S Canosa S Michaud M Graesser D Madri JA. Enhanced susceptibility to endotoxic shock and impaired STAT3 signaling in CD31-deficient mice. Am J Pathol (2005) 166(1):185–96. 10.1016/S0002-9440(10)62243-2 15632011 PMC1602311

[79] Lin F Wang N Zhang TC. The role of endothelial-mesenchymal transition in development and pathological process. IUBMB Life (2012) 64(9):717–23. 10.1002/iub.1059 22730243

[80] Xiao L Kim DJ Davis CL McCann JV Dunleavey JM Vanderlinden AK Tumor endothelial cells with distinct patterns of TGFbeta-driven endothelial-to-mesenchymal transition. Cancer Res (2015) 75(7):1244–54. 10.1158/0008-5472.CAN-14-1616 25634211 PMC4383705

[81] Gong H Lyu X Wang Q Hu M Zhang X. Endothelial to mesenchymal transition in the cardiovascular system. Life Sci (2017) 184:95–102. 10.1016/j.lfs.2017.07.014 28716564

[82] Wynn TA. Cellular and molecular mechanisms of fibrosis. J Pathol (2008) 214(2):199–210. 10.1002/path.2277 18161745 PMC2693329

[83] Yoshimatsu Y Watabe T Roles of TGF-beta signals in endothelial-mesenchymal transition during cardiac fibrosis. Int J Inflamm (2011) 2011:724080 10.4061/2011/724080 PMC3235483 22187661

[84] Chen PY Qin L Baeyens N Li G Afolabi T Budatha M Endothelial-to-mesenchymal transition drives atherosclerosis progression. J Clin Invest (2015) 125(12):4514–28. 10.1172/JCI82719 26517696 PMC4665771

[85] Zeisberg EM Tarnavski O Zeisberg M Dorfman AL McMullen JR Gustafsson E Endothelial-to-mesenchymal transition contributes to cardiac fibrosis. Nat Med (2007) 13(8):952–61. 10.1038/nm1613 17660828

[86] Zhou X Chen X Cai JJ Chen LZ Gong YS Wang LX Relaxin inhibits cardiac fibrosis and endothelial-mesenchymal transition via the Notch pathway. Drug Des Devel Ther (2015) 9:4599–611. 10.2147/DDDT.S85399 26316699 PMC4541540

[87] Kang Z Ji Y Zhang G Qu Y Zhang L Jiang W. Ponatinib attenuates experimental pulmonary arterial hypertension by modulating Wnt signaling and vasohibin-2/vasohibin-1. Life Sci (2016) 148:1–8. 10.1016/j.lfs.2016.02.017 26860892

[88] Guihard PJ Yao J Blazquez-Medela AM Iruela-Arispe L Bostrom KI Yao Y. Endothelial-mesenchymal transition in vascular calcification of Ins2Akita/+ mice. PLoS One (2016) 11(12):e0167936. 10.1371/journal.pone.0167936 27936229 PMC5148029

[89] Paruchuri S Yang JH Aikawa E Melero-Martin JM Khan ZA Loukogeorgakis S Human pulmonary valve progenitor cells exhibit endothelial/mesenchymal plasticity in response to vascular endothelial growth factor-A and transforming growth factor-beta2. Circ Res (2006) 99(8):861–9. 10.1161/01.RES.0000245188.41002.2c 16973908 PMC2810464

[90] Harikrishnan K Cooley MA Sugi Y Barth JL Rasmussen LM Kern CB Fibulin-1 suppresses endothelial to mesenchymal transition in the proximal outflow tract. Mech Dev (2015) 136:123–32. 10.1016/j.mod.2014.12.005 25575930 PMC4868094

[91] Guo Y Li P Bledsoe G Yang ZR Chao L Chao J Kallistatin inhibits TGF-beta-induced endothelial-mesenchymal transition by differential regulation of microRNA-21 and eNOS expression. Exp Cell Res (2015) 337(1):103–10. 10.1016/j.yexcr.2015.06.021 26156753 PMC4560618

[92] Chen X Cai J Zhou X Chen L Gong Y Gao Z Protective effect of spironolactone on endothelial-to-mesenchymal transition in HUVECs via Notch pathway. Cell Physiol Biochem (2015) 36(1):191–200. 10.1159/000374063 25967959

[93] Gao H Zhang J Liu T Shi W. Rapamycin prevents endothelial cell migration by inhibiting the endothelial-to-mesenchymal transition and matrix metalloproteinase-2 and -9: an in vitro study. Mol Vis (2011) 17:3406–14. 22219636 PMC3247170

[94] Correia AC Moonen JR Brinker MG Krenning G FGF2 inhibits endothelial-mesenchymal transition through microRNA-20a-mediated repression of canonical TGF-beta signaling. J Cell Sci (2016) 129(3):569–79. 10.1242/jcs.176248 26729221

[95] Chen PY Qin LF Tellides G Simons M Fibroblast growth factor receptor 1 is a key inhibitor of TGF beta signaling in the endothelium. Sci Signal (2014) 7(344):ra90 10.1126/scisignal.2005504 25249657

[96] Nagai T Kanasaki M Srivastava SP Nakamura Y Ishigaki Y Kitada M N-acetyl-seryl-aspartyl-lysyl-proline inhibits diabetes-associated kidney fibrosis and endothelial-mesenchymal transition. Biomed Res Int (2014) 2014:696475. 10.1155/2014/696475 24783220 PMC3982268

[97] Sziksz E Pap D Lippai R Beres NJ Fekete A Szabo AJ Fibrosis related inflammatory mediators: role of the IL-10 cytokine family. Mediators Inflamm (2015) 2015:764641. 10.1155/2015/764641 26199463 PMC4495231

[98] Medici D. Endothelial-mesenchymal transition in regenerative medicine. Stem Cells Int (2016) 2016:6962801. 10.1155/2016/6962801 27143978 PMC4838799

[99] Xu XB Friehs I Hu TZ Melnychenko I Tampe B Alnour F Endocardial fibroelastosis is caused by aberrant endothelial to mesenchymal transition. Circ Res (2015) 116(5):857–66. 10.1161/Circresaha.116.305629 25587097 PMC4344885

[100] Okayama K Azuma J Dosaka N Iekushi K Sanada F Kusunoki H Hepatocyte growth factor reduces cardiac fibrosis by inhibiting endothelial-mesenchymal transition. Hypertension (2012) 59(5):958–65. 10.1161/HYPERTENSIONAHA.111.183905 22392903

[101] Wu M Tang RN Liu H Pan MM Lv LL Zhang JD Cinacalcet ameliorates cardiac fibrosis in uremic hearts through suppression of endothelial-to-mesenchymal transition. Int J Cardiol (2014) 171(3):e65–9. 10.1016/j.ijcard.2013.11.105 24382406 PMC4887190

[102] Wylie-Sears J Levine RA Bischoff J Losartan inhibits endothelial-to-mesenchymal transformation in mitral valve endothelial cells by blocking transforming growth factor-beta-induced phosphorylation of ERK. Biochem Biophys Res Commun (2014) 446(4):870–5. 10.1016/j.bbrc.2014.03.014 24632204 PMC4007266

[103] Zhou H Chen X Chen L Zhou X Zheng G Zhang H Anti-fibrosis effect of scutellarin via inhibition of endothelial-mesenchymal transition on isoprenaline-induced myocardial fibrosis in rats. Molecules (2014) 19(10):15611–23. 10.3390/molecules191015611 25268717 PMC6271942

[104] Bischoff J Casanovas G Wylie-Sears J Kim DH Bartko PE Guerrero JL CD45 Expression in Mitral valve endothelial cells after myocardial infarction. Circ Res (2016) 119(11):1215–25. 10.1161/CIRCRESAHA.116.309598 27750208 PMC5215059

[105] Kanasaki K Shi S Kanasaki M He J Nagai T Nakamura Y Linagliptin-mediated DPP-4 inhibition ameliorates kidney fibrosis in streptozotocin-induced diabetic mice by inhibiting endothelial-to-mesenchymal transition in a therapeutic regimen. Diabetes (2014) 63(6):2120–31. 10.2337/db13-1029 24574044

[106] Cipriani P Di Benedetto P Ruscitti P Capece D Zazzeroni F Liakouli V The Endothelial-mesenchymal transition in systemic sclerosis is induced by Endothelin-1 and transforming growth factor-beta and may be blocked by macitentan, a dual endothelin-1 receptor antagonist. J Rheumatol (2015) 42(10):1808–16. 10.3899/jrheum.150088 26276964

[107] Groth A Vrugt B Brock M Speich R Ulrich S Huber LC. Inflammatory cytokines in pulmonary hypertension. Respir Res (2014) 15:47. 10.1186/1465-9921-15-47 24739042 PMC4002553

[108] Chen Y Yuan T Zhang H Yan Y Wang D Fang L Activation of Nrf2 attenuates pulmonary vascular remodeling via inhibiting endothelial-to-mesenchymal transition: an insight from a plant polyphenol. Int J Biol Sci (2017) 13(8):1067–81. 10.7150/ijbs.20316 28924387 PMC5599911

[109] Reynolds AM Holmes MD Danilov SM Reynolds PN. Targeted gene delivery of BMPR2 attenuates pulmonary hypertension. Eur Respir J (2012) 39(2):329–43. 10.1183/09031936.00187310 21737550

[110] Shanahan CM Inflammation ushers in calcification: a cycle of damage and protection? Circulation (2007) 116(24):2782–5. 10.1161/CIRCULATIONAHA.107.749655 18071088

[111] Evrard SM Lecce L Michelis KC Nomura-Kitabayashi A Pandey G Purushothaman KR Endothelial to mesenchymal transition is common in atherosclerotic lesions and is associated with plaque instability. Nat Commun (2016) 7:11853. 10.1038/ncomms11853 27340017 PMC4931033

[112] Yu W Liu Z An S Zhao J Xiao L Gou Y The endothelial-mesenchymal transition (EndMT) and tissue regeneration. Curr Stem Cell Res Ther (2014) 9(3):196–204. 10.2174/1574888X09666140213154144 24524794

[113] Kovacic JC Mercader N Torres M Boehm M Fuster V Epithelial-to-mesenchymal and endothelial-to-mesenchymal transition: from cardiovascular development to disease. Circulation (2012) 125(14):1795–808. 10.1161/CIRCULATIONAHA.111.040352 22492947 PMC3333843

[114] Bostrom KI Yao J Guihard PJ Blazquez-Medela AM Yao Y. Endothelial-mesenchymal transition in atherosclerotic lesion calcification. Atherosclerosis (2016) 253:124–7. 10.1016/j.atherosclerosis.2016.08.046 27615595 PMC5064862

[115] Malhotra R Burke MF Martyn T Shakartzi HR Thayer TE O’Rourke C Inhibition of bone morphogenetic protein signal transduction prevents the medial vascular calcification associated with matrix Gla protein deficiency. PLoS One (2015) 10(1):e0117098. 10.1371/journal.pone.0117098 25603410 PMC4300181

[116] Cavalera M Wang J Frangogiannis NG. Obesity, metabolic dysfunction, and cardiac fibrosis: pathophysiological pathways, molecular mechanisms, and therapeutic opportunities. Transl Res (2014) 164(4):323–35. 10.1016/j.trsl.2014.05.001 24880146 PMC4180761

[117] Lumeng CN Saltiel AR Inflammatory links between obesity and metabolic disease. J Clin Invest (2011) 121(6):2111–7. 10.1172/JCI57132 21633179 PMC3104776

[118] Kong P Christia P Frangogiannis NG. The pathogenesis of cardiac fibrosis. Cell Mol Life Sci (2014) 71(4):549–74. 10.1007/s00018-013-1349-6 23649149 PMC3769482

[119] Rizvi AA. Cytokine biomarkers, endothelial inflammation, and atherosclerosis in the metabolic syndrome: emerging concepts. Am J Med Sci (2009) 338(4):310–8. 10.1097/MAJ.0b013e3181a4158c 19726972

